# Physiological Role of Plasmacytoid Dendritic Cells and Their Potential Use in Cancer Immunity

**DOI:** 10.1155/2008/106321

**Published:** 2009-01-26

**Authors:** Jorge Schettini, Pinku Mukherjee

**Affiliations:** ^1^Department of Immunology, Mayo Clinic College of Medicine, Mayo Clinic Arizona, 13400 E. Shea Blvd, Scottsdale, AZ 85259, USA; ^2^Department of Biology, University of North Carolina at Charlotte, 9201 University City Blvd, Charlotte, NC 28223, USA

## Abstract

Dendritic cells (DCs) play a pivotal role in the control of innate and adaptive immune responses. They are a heterogeneous cell population, where plasmacytoid dendritic cells (pDCs) are a unique subset capable of secreting high levels of type I IFNs. It has been demonstrated that pDCs can coordinate events during the course of viral infection, atopy, autoimmune diseases, and cancer. Therefore, pDC, as a main source of type I IFN, is an attractive target for therapeutic manipulations of the immune system to elicit a powerful immune response against tumor antigens in combination with other therapies. The therapeutic vaccination with antigen-pulsed DCs has shown a limited efficacy to generate an effective long-lasting immune response against tumor cells. A rational manipulation and design of vaccines which could include DC subsets outside “Langerhans cell paradigm” might allow us to improve the therapeutic approaches for cancer patients.

## 1. INTRODUCTION

There is not a clear answer why tumor immunity is not effectively mounted in most tumor-bearing hosts. Early mouse studies, as well as clinical experience, indicate that the immune system can recognize and reject tumors [1–11]. On the contrary, immune-deficient mice and patients have an augmented incidence of cancer which suggests a relevant role for the immune system [12, 13]. Immunotherapeutic protocols based on these findings have been developed; however, the results are variable and limited [14–19]. As observed in melanoma and other tumors, there is an absence of specific cytotoxic T lymphocytes (CTLs) expansion in cancer patients. This suggests that tumor-antigens may not overcome the threshold on the surface of DCs needed to trigger CTL proliferation (passive factor). In addition, immunoregulatory factors are involved in downregulating T cell proliferation and inducing T regulatory cells (active factors), secreted by tumor cells [14]. Thus, DCs play a critical role in inducing and regulating the immune responses [20, 21].

DCs constitute a heterogeneous cell population, which are classified according to cluster of differentiation (CD) expression, functionality, and localization, playing a pivotal role in the control of innate and adaptive immune responses [22]. Generally, DCs' life cycle is based on a model commonly referred to as the “Langerhans cells paradigm” [23]. Immature DCs are strategically located in peripheral and interstitial spaces of most tissues, and from their location, and always in surveillance mode, DCs constitutively take up antigens from the environment, which will be associated with the MHC molecules. Coordinately, DCs mature by cessation of phagocytosis and endocytosis and move toward the draining lymphoid nodes (LNs) due to upregulation of chemokine receptor CCR7, thereby, acquiring responsiveness to a chemotactic gradient of CCL21(-Leu/-Ser) and CCL19 expressed by initial and terminal lymphatic vessels and by mature DCs, respectively [24, 25].

After arriving at the draining lymphoid nodes, DCs are able to present antigens in the context of MHC and costimulatory molecules to antigen-specific T cells. This induces a cellular immune response which drives T cells to differentiate to effectors cells [26, 27]. Moreover, DCs are important in starting adaptive and innate immunity, by activating naïve and memory B cells, natural killer, and natural killer T cells [28–31].

Due to the antigen capturing and presenting properties of DCs, ex vivo delivery of tumor-antigen to DCs has been used as a strategy to guarantee successful antigen presentation to T cells [14]. However, the efficacy of this approach to therapeutic vaccination has been limited in both preclinical and clinical settings [19, 32]. This suggests that we need to better understand and refine the parameters to establish the optimal conditions for vaccination against cancer.

Recent progress in the identification of distinct DC subsets has been done. Analysis of the DC population in several lymphoid organs has shown a considerable heterogeneity, where some subsets of DCs follow the “Langerhans cell paradigm”, but not all of them [33, 34]. Unfortunately, the heterogeneity of the human DC network is poorly understood compared with the mouse DC network. At present, there are two main pathways of differentiation in mouse DCs. The myeloid pathway generates two subsets: Langerhans cells and interstitial DCs, whereas the lymphoid pathway generates plasmacytoid DCs (pDCs) [22, 28, 35]. In contrast to the many studies in mouse DCs, there are very few studies on mature human DCs from tissue. Human blood DCs are heterogeneous in their expression of markers, but this may reflect differences in the activation or maturation states of DCs rather than separate lineages [36]. However, from in vitro studies, it is possible to deduce pathways of human dendritic cell development. Similar to mouse DCs, the myeloid pathway in humans generates Langerhans cells and interstitial DCs. Blood monocytes, named precursors DC1 (pDC1), are the most commonly used precursor cells for generating human DCs in culture. In the presence of GM-CSF and IL-4, pDC1 can generate DCs called DC1. Maturation of these cells is achieved by stimulating cytokines or microbial products [22, 37–39]. The human lymphoid pathway also generates pDCs, termed pDC2. These cells are type I IFN producing cells (IPCs) and they were discovered before their mouse counterparts. The pDC2 responds to viral and microbial stimuli by producing type I IFNs [35]. Both human and mouse pDCs can be maturated with bacterial stimuli or viruses. Upon maturation, human pDC2, named DC2, lacks typical myeloid markers, such as its precursor, but displays the characteristic of mature DCs [40, 41].

Although most studies have focused on the role of pDCs in antiviral immunity, several new lines of evidence have suggested that pDCs are also involved in tumor immunity, as well as in promoting peripheral tolerance [42–47]. Interestingly, pDCs can synthesize large amount of functional indoleamine 2,3-dioxygenase (IDO), which requires autocrine release of type I IFN, upon Toll-like receptor-9 (TLR9) and CD200R ligands stimulation. IDO secretion by pDCs promotes T-cell death at T-cell areas of secondary lymphoid organs. Notably, through the upregulation of inducible T-cell costimulator ligand (ICOSL), pDCs have the ability to generate regulatory T cells [48, 49]. Gathering together, this evidence suggests that pDCs represent a key effector cell in both innate and adaptive immunity regulation [35, 50–53]. In this review, we focus on the characterization, physiology, and potential roles of pDCs in the antitumor responses.

## 2. DIFFERENTIATION AND TRAFFICKING PATTERNS OF pDCs

The growth factor fms-like tyrosine kinase 3 ligand (FLT3-L) has been described as a key differentiation and trafficking factor for human and mouse pDCs from hematopoietic progenitor cells (HPCs). FLT3-L injection in humans causes an increase of both myeloid DCs (mDCs) and pDCs in the blood. In mice, FLT3-L injection induces the generation of mDCs and pDCs in blood, lymphoid tissues, liver, and lung [54–59]. In vitro, mDC and pDCs can be generated from FLT3-L-supplemented BM culture system [60, 61]. Recently, Fancke et al. have also shown that M-CSF is capable of driving pDCs from bone marrow precursor cells in vitro and in vivo [62].

pDCs account for less than 1% of total peripheral blood mononuclear cells (PBMCs) and can be isolated through removal of lineage-positive cells and CD123^+^ (IL-3R). The identification of two markers on human (BDCA-2 and BDCA-4) and one in the mouse (PDCA-1) has facilitated the isolation of pDCs from PBMC or lymphoid organs by positive selection with magnetic beads coupled with specific monoclonal antibodies [63, 64].

In human and mice, pDCs have been found circulating in the blood and cord blood of neonates [65–67]. Interestingly, human pDCs have been found in fetal liver, thymus, and bone marrow suggesting that pDCs develop from CD34^+^ human stem cells (HSCs) within these primary lymphoid tissues [68]. Moreover, pDCs can be located in lymphoid nodes, spleen, tonsils, and Peyer's patches.

Similar to B and T cell migration patterns, pDCs leave the bone marrow and migrate into the T cell rich areas of the secondary lymphoid tissues, through high-endothelial venules (HEVs) in the lymph nodes, mucosa-associated lymphoid tissues, and through marginal zones of the spleen under steady-state conditions [69–73]. This unique migration pattern of pDCs among DCs appears to be connected with their expression of CD62L and CCR7, which allows the pDCs ligate L-selectin ligands expressed by HEV and chemokines CCL19 and CCL21 expressed by HEVs and stromal cells within the T-cell rich areas, respectively [73, 74].

The expressions of chemokine receptors on circulating blood mDCs and pDCs are similar. However, the level of CCR5, CCR7, and CXCR3 expressions is clearly divergent in these two subsets, being higher on pDCs than on mDCs [74]. Among these two subsets, pDCs are also the only to migrate in response to the homeostatic chemokine SDF-1/CXCL12, the ligand of CXCR4, which is expressed on dermal endothelial cells, in HEVs of lymphoid nodes, and in malignant cells [44]. This evidence suggests that pDCs may reach lymph nodes using CXCR4, and also explains their fundamental localization in the secondary lymphoid organs [70].

Interestingly, human pDCs have been found to infiltrate primary and malignant melanoma, head and neck carcinoma, ovarian carcinoma, and breast cancer [42–46, 75], as well as cutaneous inflammatory lesions, which may be dependent on their ability to express CLA, which binds to E-selectin on dermal endothelial cells and may enhance their recruitment to the inflammatory site [76].

## 3. ACTIVATION OF PLASMACYTOID DCs

Virtually, all cell types are able to produce type I IFNs in response to viral exposure. The amount, kinetics, and types of IFN will depend on the cell type. However, pDCs are considered the professional type I IFN producing cells [35]. pDCs can produce 100–1000 times more type I IFN than the other blood cell types upon activation [35], or the equivalent of 10 pg/cell [77]. Myeloid DCs can also secrete type I IFN in response to RNA viruses, but less efficiently than pDCs [78].

It is important to note that not all viruses can activate pDCs to produce IFNs. Also, pDCs do not require to be infected to secrete type I IFN [79, 80]. Once secreted, type I IFNs induce MxA, an IFN*α*-inducible intracellular protein [75], oligoadenylate synthetase, and double-stranded RNA-(dsRNA-)-dependent protein kinase (PKR). Together, these proteins have the biological role in inducing cellular resistance by blocking viral replication, and, therefore, viral spread [81].

Moreover, type I IFN modulates several aspects of the immune response, including pDC survival, mDCs differentiation [82], modulation of Th1 and CD8^+^ T-cell responses, cross-presentation and cross-priming independent of CD4^+^ T helper cells [83], upregulation of MHC and costimulatory molecules, activation of NK cells, and induction of primary antibody responses [84].

pDC activation with pathogens or oligodeoxynucleotides (ODNs) with multiple unmethylated CpG dinucleotides induces the secretion of several other cytokines and chemokines, such as TNF*α*, IL-1, and IL-6. In mouse, but not in humans, pDCs have the capacity to synthesize bioactive IL-12, although this capacity still remains controversial [85–87]. Virally, stimulated pDC produces chemokines, such as CCL3 (MIP-1a), CCL4 (MIP-1b), CCL5 (RANTES), CXCL8 (IL-8), and CXCL10 (IP-10) which stimulate Th1, and NK cells homing to site of infection through IP-10 and CCL4, respectively [88, 89].

## 4. REGULATION OF TYPE I IFN SYNTHESIS ON pDCs

This unique subset of DCs can secrete type I IFNs faster than other cells to a wider range of viral and nonviral stimuli. Moreover, pDCs express a broader profile of IFNA genes than other antigen-presenting cells (APCs). In humans, the type I IFN family consists of 13 IFN*α* subtypes, one IFN*β*, one IFN-*ω*, one IFN-*κ*, and one IFN-*τ*. IFN*α*1 is the major subtype expressed by pDCs, but other subtypes are also secreted, including IFN*α*2, -*α*5, -*α*8, -*α*10, and -*α*14 and a recently described family of IFN*λ*1-3 (also named IL-29, IL-28A, and IL-28B, resp.) [90, 91].

What makes pDCs synthesize type I IFN faster than other cells? Recently, it has been shown that transcription factors of the family of interferon regulatory factors (IRFs) play an important role in the regulation of interferon gene transcription. Nine mammalian IRF family members have been identified to guide the induction of IFN production, as well as to regulate and differentiate various cells types [92]. Expression of IRF-3 supports induction of IFN*β* and IRF-5 or IRF-7 is sufficient to stimulate IFN*α* genes expression. Unlike other cells, pDCs have been shown to express constitutively higher levels of IRF-5, -7, and -8 mRNA, which might explain why this particular subset of DCs secrete faster and large quantities of type I IFNs than other cell types [93, 94].

## 5. DIFFERENTIAL EXPRESSION AND FUNCTION OF TLRs IN pDCs

This unique ability of pDCs to secrete large amounts of type I IFN depends on cellular receptors able to sense several types of nucleic acid. TLR is a family of 11 pattern recognition receptors (PRRs) which mediate the recognition of many pathogens through the detection of distinct pathogen-associated molecular patterns (PAMPs) [95, 96].

pDCs and mDCs each has a different TLR expression profile. In humans, mDCs can express TLR-1, -2, -3, -4, -5, -7, and -8, while pDCs express mainly TLR7 and -9 [97, 98]. Uniquely, TLR-7, -8, and -9 detect PAMPs in endosomal/lysosomal compartments followed by acidification [99, 100]. Transcriptional regulation of IFN*β* and IFN*α* genes on pDCs is controlled mainly by IRF-3 and IRF-5/7. IRF-3 can be activated by TLR-3 and TLR-4, but there is no evidence of this pathway on pDCs. Instead, IRF-7 has a constitutively high expression in pDCs and it is recruited by myeloid differentiation primary response gene 88 (MyD88) through the adaptor molecule TRAF6 when TLR-7 or -9 is triggered [101].

Many studies have shown that exposure to synthetic TLR-7 or -9 agonists (e.g., imidazoquinoline, CpG ODN) induces pDCs to secrete IFN*α* and proinflammatory cytokines (IL-8 and TNF*α*), maturation, which heighten their T-cell stimulatory capacity [97, 102–104].

Interestingly, endogenous antigens, such as DNA from necrotic cells, may be taken up by pDCs and signal through TLR-9 in autoimmune diseases [105]. TLR-9 agonist has a therapeutic potential and it has been used to induce innate and adaptive immune responses. Synthetic TLR-9 agonists are currently being tested in multiple phase II and phase III human clinical trial as adjuvants to cancer vaccine and in combination with conventional chemotherapy and others protocols [106–108].

## 6. pDCs CAN LINK INNATE AND ADAPTIVE IMMUNITY VIA TYPE I IFNs

There are abundant studies in human and mice showing the importance of type I IFN to regulate inflammation and link innate and adaptive immunity [113–115]. IFN*α* and -*β* are considered as important components of innate immunity together with their well-known antiviral activity [114]. Type I IFN released by human pDCs activates NK cell cytolytic activity, and also induces IFN*γ* production in NK cells through IL-12 secretion [116, 117]. Although with different molecular mechanisms in human and mice, type I IFN secreted by pDCs, upon stimulation, can affect T-cell functions. Thus, activated pDCs can induce T cells to make IL-10 and IFN*γ* [113, 118], and also induce a Th1 polarization [119]. It has also been reported that type I IFN can induce early activation markers (CD69) on T cells, long-term survival [120], and generation of a long-term antitumor immune response [121]. Recently, several studies have provided important evidence for a role of type I IFN in the differentiation of the Th1 subset [122], in the generation and activity of CTLs, as well as in supporting in vivo proliferation and survival of T cells [123, 124]. Altogether, these studies have led to the recognition of an important role of this cytokine in linking innate with adaptive immunity [115, 125].

On the other hand, murine pDCs can also inhibit certain mDCs functions. Upon infection, mice pDCs are the primary source of IFN*α* and IL-12, and type I IFNs they produce inhibit the synthesis of IL-12 from mDCs, a critical immunostimulatory cytokine of the T-cell-mediated immunity [79]. In human, the production of IL-12 by pDCs is still controversial, but some studies claimed the contrary [98, 126].

Interestingly, pDCs are critical for the generation of plasma cells and antibody responses. It appears that the depletion of pDCs from human blood abrogates the secretion of IgGs in response to viral infection. Furthermore, activated pDCs can induce activated B cells to differentiate plasma cells. Through Type I IFN and IL-6 secreted by pDCs, B cells are induced to develop into plasmablast and differentiate into antibody-secreting plasma cells [29].

## 7. PLASMACYTOID DCs AND THEIR ROLE IN CANCER IMMUNITY

Before the maturation of pDCs, they have a poor T-cell stimulation capacity. Early experiments reported that CD40L in combination with IL-3-stimulated pDCs develop into a functionally distinct DCs type that promotes the development of IL-4-secreting Th2 cells [40]. Also, pDCs can prime Th1 or Th0 allogeneic responses [118, 127, 128]. Furthermore, pDCs mature following exposure to influenza virus and exhibited an equivalent efficiency to expand the repertoire of anti-influenza virus cytotoxic T lymphocytes and Th1 CD4^+^ T cells [104, 129].

It is clear now that immature mDCs and pDCs infiltrate solid tumor and lack the ability to induce T-cell activation [75]. However, they still present tumor antigens and induce IL-10-producing CD4^+^/CD25^+^ regulatory T cells that inhibit antitumor immunity [130]. Nevertheless, using an anti-IL-10 mAb and CpG ODN, it is possible to induce a robust antitumor CTL response and tumor rejection in vivo [111]. Recently, murine pDCs have been described to have the ability to elicit in vivo, in naïve mice, an antigen-specific CD8^+^ T cell response against endogenous antigens, as well as exogenous peptides, but not against exogenous antigens, and were capable of protecting mice from tumor challenge [131].

It has also been reported that human tumor antigens pulsed pDCs in vitro can prime IFN*γ*-secreting melanoma-specific CTLs [42]. Synergy among DC subsets has not been fully explored in the development of antitumor immunity. An interesting study has shown that immunizations with a mixture of matured pDCs plus mDCs resulted in increased levels of antigen-specific CD8^+^ T cells and an enhanced antitumor response compared with immunization with either dendritic cell subset alone [109]. Altogether, these studies suggest that it is possible to re-establish and/or maximize an antitumor immune response when pDCs are taken in the regimen [132–137] (Table 1).

## 8. CLINICAL SIGNIFICANCE OF pDCs

There is evidence that pDCs are located in several types of tumors: head and neck cancer, ovarian cancer, primary melanoma cancer, and breast cancer [42–46, 75]. Secreted factors by tumor cells may inhibit pDCs function, such as TGF*β*, vascular endothelial growth factor *β* (VEGF*β*), and IL-10.

On the contrary, other studies have reported that pDCs and tumor-infiltrating DC (TIDC) are functional and fully competent APCs. Isolation of TIDC showed an intermediate maturation phenotype and the capacity to take up particles, as well as produce IL-12 and maintain its migratory capacity. Infiltrating pDCs are capable of producing IFN*α*, as well as inducing complete regression or significant reduction of melanomas after a topically treatment of imiquimod (a small synthetic immune response modifier recognized by TLR7) [46, 110, 138, 139]. In addition, intratumoral stimulation of pDCs with TLR7 and -9 agonists has been successfully used in the clinic to treat basal cell carcinoma, human papillomavirus-infected warts, and condylomata accuminata [140, 141]. TLR signaling on pDCs can be used to induce type I IFNs and possibly protect DCs from tumor-derived inhibitory factors (such as VEGF*β* or IL-10), as well as support T-cell survival, therefore, improving vaccination efficacy [112, 142–147].

Thus, it will be critical to evaluate if stimulation of pDCs may overcome tumor-mediated inhibitory effects and can enhance a local antitumor immunity.

## 9. CONCLUSIONS

DCs are a heterogeneous cell population, where plasmacytoid dendritic cells (pDCs) are a unique subset capable of secreting high levels of type I IFNs. It has been demonstrated that pDCs can coordinate events during the course of viral infection, atopy, autoimmune diseases, and cancer. Therefore, pDCs as a main source of type I IFN is an attractive target for therapeutic manipulations of the immune system to elicit a powerful immune response against tumor antigens in combination with others therapies.

A rational manipulation and design of vaccines which could include DCs subsets outside “Langerhans cell paradigm” might allow us to improve the therapeutic approaches for cancer patients.

## Figures and Tables

**Table 1:** 

Tumor	System	DC source	Protocol	References
EG7 T-cell lymphoma	Murine	Expanded in vivo (FLt3L), and sorted from BM	CpG-activated OVAp-pulsed pDCs/mDCs	Lou et al. [109] (2007)

K17-35-OVA melanoma	Murine	Isolated TIDCs from K17-35 melanoma	OVA-pulsed TIDCs	Preynat-Seauve et al. [110] (2006)

C26 colon Carcinoma	Murine	Isolated TIDCs from C26 tumor	TIDCs activated with CpG + anti-IL-10R (i.p.)	Vicari et al. [111] (2002)

M3 Melanoma	Murine	—	Topical application Imiquimod	Palamara et al. [46] (2004)

Melanoma cell lines	Human	Sorted from PBMC	pDCs activated with CD40L-transfected J558	Salio et al. [42] (2003)

Melanoma stage IIIb/c, IV	Human	—	CpG-7909 (s.c.) (ProMune)	Pashenkov et al. [112] (2006)

DCs, dendritic cells; pDCs, plasmacytoid DCs; BM, bone-marrow; OVAp, OVA peptide; TIDCs, tumor-infiltrating DCs (myeloid and plasmacytoid); PBMC, peripheral blood mononuclear cells.
